# Translation, cultural adaptation and validation of the Stapesplasty Outcome Test 25 (SPOT-25) for measurement of disease-specific health-related quality of life in Dutch otosclerosis patients: a prospective study

**DOI:** 10.1007/s00405-025-09353-5

**Published:** 2025-05-08

**Authors:** Esther E. Blijleven, Maaike Jellema, Joeri Buwalda, Raphael J. B. Hemler, Huib F. van Waegeningh, Robert J. Stokroos, Hans G. X. M. Thomeer, Inge Wegner

**Affiliations:** 1 Department of Otorhinolaryngology, Head and Neck Surgery, University Medical Center Utrecht, P.O. Box 85500, 3508 GA Utrecht, The Netherlands; 2 Brain Center, University Medical Center Utrecht, Utrecht, The Netherlands; 3 Department of Otorhinolaryngology, Deventer Hospital, Deventer, The Netherlands; 4 Department of Otorhinolaryngology, Gelre Hospital, Apeldoorn, The Netherlands; 5 Department of Otorhinolaryngology, Tergooi Hospital, Hilversum, The Netherlands; 6 Department of Otorhinolaryngology, Head and Neck Surgery, University Medical Center Groningen, Groningen, The Netherlands

**Keywords:** Otology, Otosclerosis, Stapes surgery, Hearing loss, HRQOL, PROMs

## Abstract

**Purpose:**

To translate and culturally adapt the SPOT-25 to the Dutch language and validate the Dutch SPOT-25 in a Dutch population of otosclerosis patients undergoing primary stapes surgery.

**Methods:**

A multicenter prospective validation study was performed between November 2018 and May 2024. The translation into Dutch and validation process of the SPOT-25 was performed according to the COSMIN guidelines. Patients were asked to complete the SPOT-25 and Glasgow Health Status Questionnaire (GHSQ) preoperatively, the SPOT-25, GHSQ and Glasgow Benefit Inventory six to eight weeks postoperatively and the SPOT-25 eight to ten weeks postoperatively. Healthy controls were asked to complete the translated SPOT-25 once. Preoperative and postoperative audiometric results were also obtained. The evaluated measurement properties included construct validity, measurement invariance, discriminative validity, reliability and responsiveness of the translated SPOT-25.

**Results:**

Hundred and fifteen patients and 50 healthy controls were analyzed. Analyses of the translated SPOT-25 showed adequate construct validity, discriminative validity, reliability and responsiveness. The SPOT-25 scores were strongly correlated with the GHSQ score. The internal consistency and test–retest reliability were good as Cronbach’s alpha and intraclass correlation coefficients were higher than 0.70. The four-factor model fitted best in our population of otosclerosis patients; however the results indicated a mediocre fit between the model and the data.

**Conclusion:**

The Dutch SPOT-25 showed good validity, reliability and responsiveness and can be implemented as an additional outcome measure to improve otosclerosis research and clinical practice.

**Supplementary Information:**

The online version contains supplementary material available at 10.1007/s00405-025-09353-5.

## Introduction

Otosclerosis involves abnormal bone overgrowth predominantly around the oval window region which can cause fixation of the stapes footplate, resulting in acquired conductive hearing loss (often combined with sensorineural hearing loss), vertigo and/or tinnitus [1]. The treatment of conductive hearing loss consists of the use of hearing aids or surgery. During surgery, part of the stapes is replaced with a prosthesis. Current guidelines recommend reporting the pure-tone audiometric thresholds and/or speech discrimination scores to assess postoperative outcomes of stapes surgery [2, 3]. Surgical success is often defined as a postoperative air–bone gap of 10 dB or less, or 20 dB or less (PTA: Pure Tone Average). However, postoperative patient-reported hearing disability correlates better with mental health and health-related quality of life, than with postoperative audiometric results, including postoperative air–bone gap or air conduction levels [4, 5]. The psychological effects of stapes surgery and its impact on health-related quality of life can be measured using patient-reported outcome measures. These measures include disease-specific and generic questionnaires of quality of life and are used in clinical practice and research to gain insight from the patient’s perspective on their physical and social functioning, mental health and well-being [6].

In our opinion, to estimate the subjective benefit of stapes surgery, health-related quality of life measurements should be implemented as an additional outcome measure after stapes surgery. The Stapesplasty Outcome Test 25 (SPOT-25) is the only health-related quality of life measurement that has been validated for use in otosclerosis patients. It has been validated for use in the German, Greek, French and Danish language and culture [7–11]. Due to the lack of a Dutch health-related quality of life questionnaire for Dutch otosclerosis patients, the aim of this study was to translate and culturally adapt the SPOT-25 to the Dutch language and validate the Dutch SPOT-25 in a Dutch population of otosclerosis patients undergoing primary stapes surgery.

## Materials and methods

### Study design and ethics approval

A prospective validation study was performed in a tertiary care center and three secondary care centers between November 2018 and January 2024. The study was performed in two stages. Firstly, the SPOT-25 was translated from German to Dutch and culturally adapted. Secondly, the translated SPOT-25 was validated in a group of Dutch otosclerosis patients and a healthy control group. The protocol for this study was approved by the Institutional Review Board of the University Medical Center Utrecht (protocol 18–768/C; V.1, November 2018) and previously published [12].

### Translation and cultural adaptation of the SPOT-25

With permission from the lead author of the SPOT-25, the original SPOT-25 developed by Lailach et al. was translated and culturally adapted from German to Dutch.

**Fig. 1:**
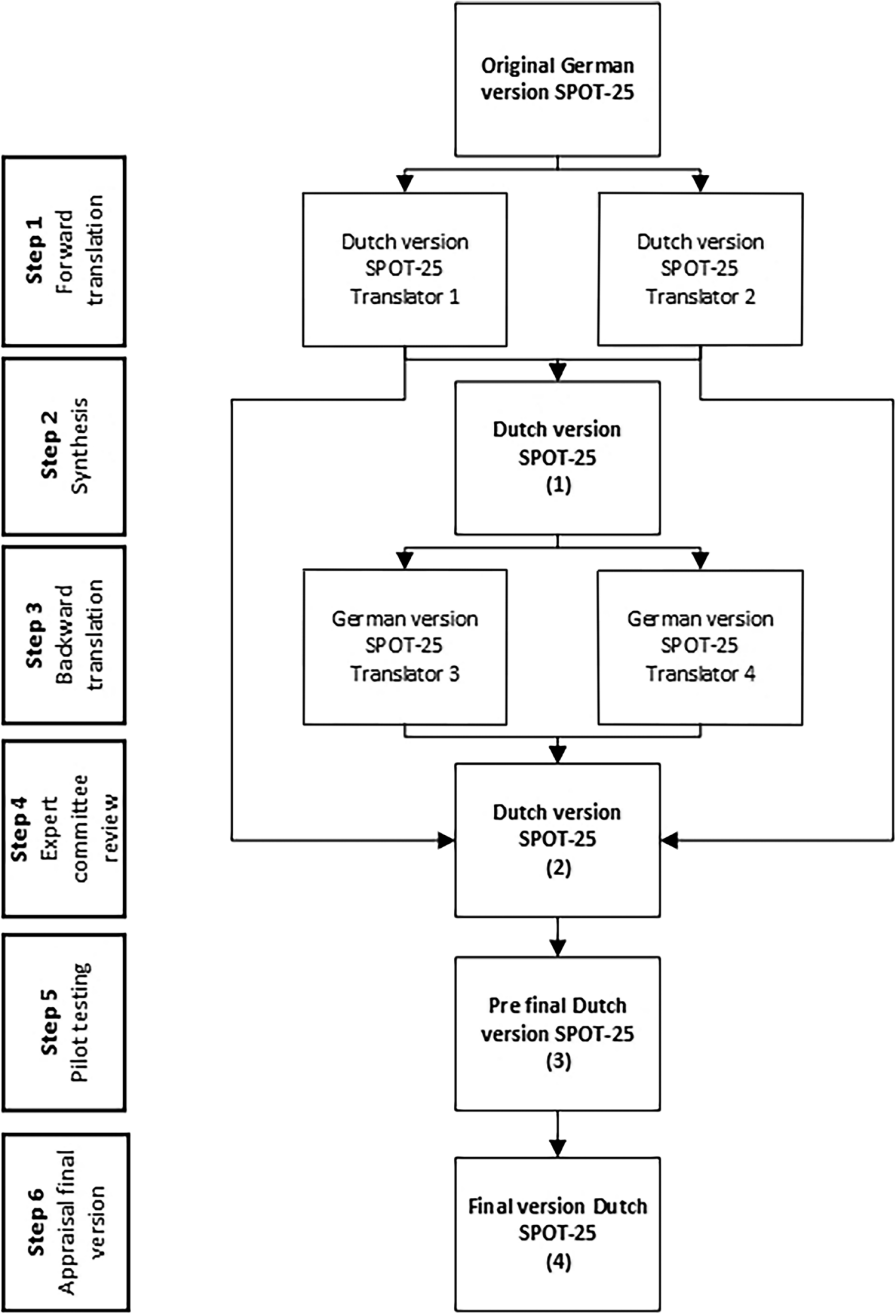
Process of translation and cultural adaptation

The translation and cultural adaptation were performed in a six-step process in accordance with the guideline of Beaten et al. and the COSMIN guideline, which is shown in Fig. 1 [13–15].

#### Step 1: Forward translation

The original SPOT-25 was translated forward from German to Dutch by two bilingual translators with the Dutch language as their mother tongue. Forward translator 1 was an otorhinolaryngologist, who was aware of the concept of the questionnaire. Forward translator 2, a naive translator, had no clinical or medical background and was unfamiliar with the concept of the questionnaire. Both translators independently made a written report of the completed translation.

#### Step 2: Synthesis

The forward translations were combined into one translation during a consensus meeting between the two translators and the researchers (EB, HGXM, IW). All issues about differences in wording and phrasing were discussed and decisions about these differences were made by consensus. The synthesis process was documented.

#### Step 3: Backward translation

The forward translated SPOT-25 was backward translated from Dutch to German by two bilingual translators with the German language as their mother tongue. Both backward translators did not have a medical background and were blinded to the original German version of the SPOT-25.

#### Step 4: Expert committee review

All four translators and the researchers (EB, HGXM, IW) reviewed the four translations. The backward translations were compared to the original German version of the SPOT-25. The expert committee reached consensus on the discrepancies between the translations and composed a prefinal version of the translated SPOT-25. The issues and rationale of each decision were documented.

#### Step 5: Pretesting

The prefinal version of the translated SPOT-25 was pilot tested in a series of 15 otosclerosis patients to check the relevance of each item and the comprehensiveness of the translated SPOT-25. After completing the translated SPOT-25 preoperatively, the patients were asked by an interviewer (EB) about the wording and clarity of the questions. The data was analyzed by the researchers to confirm face validity of the translated SPOT-25.

#### Step 6: Appraisal of the final version by the developer

The final version of the translated SPOT-25 was composed by consensus of the expert committee, after reviewing the results of the pretesting. If necessary, the adapted questionnaire was re-tested in a new set of 15 patients.

### Participants

Adults aged 18 years or older, who underwent primary stapes surgery for otosclerosis were included. Healthy participants aged between 30 and 60 years without a history of ear diseases (apart from uncomplicated acute otitis or otitis media with effusion in childhood) or previous middle ear surgery (apart from the placement of ventilation tubes in childhood) were included in the control group. All participants had a good understanding of the Dutch language and were willing and able to fulfill the questionnaires. A total of 130 otosclerosis patients and 50 healthy controls were included.

### Procedures

Validation of the translated SPOT-25 entailed three measurement moments in the patient group: preoperatively, six to eight weeks postoperatively and eight to ten weeks postoperatively. There was a two-week time window for the two postoperative measurements because, in clinical practice, the first postoperative follow-up appointment is scheduled within a two-week window. The translated SPOT-25 and the Glasgow Health Status Questionnaire (GHSQ) were completed preoperatively during the patients’ preoperative visit to the outpatient clinic. Pure-tone audiometric thresholds and speech discrimination scores were also obtained during this visit. The translated SPOT-25, the GHSQ and the Glasgow Benefit Inventory (GBI) were completed six to eight weeks after surgery during the patients’ postoperative visit to the outpatient clinic and pure-tone audiometric thresholds and speech discrimination scores were also obtained during this visit. The last measurement of the translated SPOT-25 was performed at home at eight to ten weeks postoperatively. The questionnaires were sent by post or e-mail using the UMCU-endorsed system Castor Electronic Data Capture (Amsterdam, the Netherlands).

The healthy control group completed the translated SPOT-25 once. The questionnaire was sent to the healthy controls by post.

### Outcome measures

Health-related quality of life was measured using three questionnaires: the translated SPOT-25, GHSQ and GBI. The translated SPOT-25 measures four domains (‘hearing function’, ‘tinnitus’, ‘mental condition’ and ‘social restrictions’) and the total score ranges from 0 (slightest restriction) to 100 points (biggest restriction). The GHSQ measures three domains (‘general’, ‘social support’ and ‘physical health’) and the total score ranges from 0 (high health status) to 100 points (low health status). The GBI measures the same three domains as the GHSQ and the total score ranges from -100 (maximum negative benefit), through 0 (no benefit), to + 100 points (maximum benefit).

Pure-tone audiometric thresholds and speech discrimination scores at 60 or 65 dB were routinely measured. The pre- and postoperative air-conduction and bone-conduction thresholds at 0.5, 1, 2 and 3 kHz, and the corresponding air-bone gaps were averaged. Thresholds at 3 kHz are not routinely measured in the Netherlands, therefore we interpolated 3 kHz thresholds by averaging the thresholds at 2 and 4 kHz. Details regarding the outcome measures were reported in the published protocol [12].

### Validation of the translated SPOT-25

The COSMIN-methodology was used to assess the construct validity, measurement invariance, discriminative validity, reliability and responsiveness of the translated SPOT-25 [13, 14]. The definitions of these terms were explained in the published protocol [12]. Means and standard deviations (SDs) were calculated for continuous variables that were normally distributed, while medians and ranges were used for variables that were not normally distributed. Categorical variables were summarized using frequencies and percentages. The statistical analyses were performed using IBM SPSS Statistics 29.0.2.0 (IBM Corp., Armonk, NY, USA) and R statistical computing.

#### Construct validity

Construct validity was assessed by hypothesis testing using Pearson correlation coefficients. Hypotheses were formulated regarding the expected relationship between the translated SPOT-25 and the GBI, GHSQ and audiometric results and can be found in the published protocol [12]. Whenever Pearson correlation coefficients were calculated; strong, good, moderate and weak correlations were defined as correlation coefficients of more than 0.70, between 0.50 and 0.70, between 0.30 and 0.50 and less than 0.30 respectively.

#### Measurement invariance

We tested for measurement invariance using confirmatory factor analysis. We tested a one-factor model, a two-factor model of hearing function/tinnitus and mental/social factors and a three-factor model of hearing function/tinnitus, mental factors and social factors. To assess the fit of these models to the data, we used the comparative fit index (CFI), the root mean square error of approximation (RMSEA) and the standardized root mean square residual (SRMR). Models were considered to fit well if they had a CFI close to 0.95 or higher, a RMSEA close to 0.06 or lower and a SRMR close to 0.08 or lower. We expected that the original four-factor model (hearing function, tinnitus, mental conditions and social restrictions) had an adequate fit in our population of otosclerosis patients.

#### Discriminative validity

Baseline age and gender were compared between patients and healthy controls using a non-parametric Mann–Whitney U test, because the data was not normally distributed. A non-parametric Mann–Whitney U test was also used to assess divergent validity. A two-sided p-value of < 0.05 was considered statistically significant.

#### Reliability

Cronbach’s alpha was calculated to gauge internal consistency. The range of acceptable Cronbach’s alpha values was 0.70–0.90. Intraclass correlation coefficients (ICCs) based on a two-way random effect model were used to assess the absolute agreement between the two postoperative measurements. ICC values of 0.70 or higher were considered to indicate good test–retest reliability.

#### Responsiveness

Responsiveness was assessed by hypothesis testing using Pearson correlation coefficients. Hypotheses were formulated regarding the expected relationship between the translated SPOT-25 and the change scores of the GHSQ and audiometric results and these hypotheses can be found in the published protocol [12]. Strong, good, moderate and weak correlations were defined as Pearson correlation coefficients of more than 0.70, between 0.50 and 0.70, between 0.30 and 0.50 less than 0.30 respectively.

### Missing data

We investigated reasons for missing data. Data was considered missing at random. We considered deleting items with a large percentage of missing data (> 15%). This was not necessary because missing data did not exceed 15% in any of the items. Missing data were handled using multiple imputation. Ten imputed datasets were generated and analyses were conducted using pooled estimates derived from these datasets. If pooled estimates could not be calculated in SPSS, the results were obtained by averaging the values across all 10 imputed datasets.

## Results

### Study population

Hundred and thirty patients who underwent primary stapes surgery and 50 healthy controls were included between November 2018 and January 2024. Fifteen patients were excluded because they only completed the preoperative measurement. A total of 115 patients completed the preoperative and at least one postoperative measurement. 15 patients did not complete the third measurement. The mean age was 48 years (SD 11, range 25–72) in the patient group versus 45 years (SD 8, range 30–60) in the healthy control group. In the patient group 64% of the participants were female compared to 58% in the healthy control group. We found no statistically significant difference in age and gender between the two groups (p-value > 0.05).

### Translation and cultural adaptation of the SPOT-25

#### Forward translation

Two forward translations were performed. Acceptable Dutch translations were obtained for most items. During the consensus meeting special attention was paid to the comprehension of the answer options and question 21 and 25. It was difficult to find a conceptual equivalent for *‘publieke activiteiten’* (‘public activities’) in question 21. Finally, we chose the word ‘*dagelijkse beslommeringen’* (‘daily activities’) because the word* ‘publieke activiteiten’* (‘public activities’) is not ordinarily used in Dutch. Question 25 and the answer options were not translated literally but slightly paraphrased for better comprehensibility. *‘Hooggradig probleem’* (‘high level problem’) was changed to *‘ernstig probleem’* (‘serious problem’) *and ‘slechter kan het niet worden’* (‘it could not get any worse’*)* was changed to *‘zeer ernstig/ergst denkbaar probleem’* (‘very serious problem/worst problem imaginable’). *‘Algeheel oordeel’* (‘overall judgment’) in question 25 was changed to *‘algemeen oordeel’* (‘general judgment’). The researchers and the two forward translators reached consensus on all 25 questions.

#### Backward translation

The two backward translations corresponded well. During the consensus meeting of the expert committee, questions 2 and 16 were changed, because the new Dutch translation of these questions was more literal. The word *‘dof’* (‘dull’) in question 2 was changed to *‘gedempt’* (‘muffled’) and the word* ‘beschamend’* (‘embarrassing’) in question 16 was changed to *‘pijnlijk’* (‘painful’). An example of the translation process of one question is presented in Appendix 1. The expert committee reached consensus on all 25 questions and composed a prefinal Dutch version of the SPOT-25.

#### Content validity

The prefinal Dutch version of the SPOT-25 was pilot tested in 15 otosclerosis patients to confirm content validity. Face-to-face evaluation showed that patients found the questions understandable and that answering the questions was not considered time-consuming. The interviews showed that the patients differed in their interpretation of whether the questions should be answered based on their hearing experience with or without hearing aid use. During pilot testing, all patients decided for themselves whether their answers were based on their hearing experience with or without the use of hearing aids. Based on these findings, the introductory text of the questionnaire was changed after pilot testing. The researchers added a sentence stating that patients should answer the questions based on their hearing experience with hearing aids if they used one. No questions were revised after pilot testing, so re-testing was not necessary. The face validity was subjectively assessed by three authors (EB, HT, IW), who agreed that the SPOT-25 provides a valid assessment of the quality of life in otosclerosis patient by evaluating different situations that patients with hearing loss may encounter. The original and the translated questions of the SPOT-25 are presented in Appendix 2.

### Validation of the translated SPOT-25

#### Construct validity

All correlation coefficients between the postoperative SPOT-25 scores, postoperative GHSQ and GBI scores and pure-tone audiometric results are shown in Table 1. The total score of the SPOT-25 and the subscores hearing function, mental condition and social restrictions were strongly correlated with the total score of the GHSQ. The tinnitus subscore correlated moderately with the GHSQ (r =  − 0.57). All SPOT-25 scores correlated weakly with the GBI, except for the tinnitus subscore, which did not correlate with the GBI total score. Furthermore, not all audiometric results correlated with the SPOT-25 scores. Only good and moderate correlations were found between the SPOT-25 scores and postoperative air conduction.

**Table 1:** Construct validity

SPOT-25	Questionnaires		Audiometric results				
	GHSQ Total score	GBI Total score	Mean AC		Mean ABG		ABG closure ≤ 10 dB
			Postoperative	Gain	Postoperative	Gain	
Total score	** − 0.80**	** − 0.28**	**0.50**	** − 0.32**	**0.23**	** − **0.18	** − 0.23**
Hearing function	** − 0.72**	** − 0.27**	**0.55**	** − 0.36**	**0.21**	** − **0.18	** − 0.21**
Tinnitus	** − 0.57**	** − **0.18	0.19	** − 0.22**	**0.19**	** − 0.27**	** − **0.14
Mental condition	** − 0.74**	** − 0.25**	**0.39**	** − 0.23**	0.17	** − **0.10	** − 0.23**
Social restrictions	** − 0.71**	** − 0.24**	**0.44**	** − 0.22**	**0.21**	** − **0.10	** − 0.20**

Data is expressed as Pearson correlation coefficients. Correlation coefficients printed in bold were statistically significant with a p-value < 0.05

*ABG* air–bone gap, *AC* air conduction, *GBI* glasgow benefit inventory, *GHSQ* Glasgow Health Status Questionnaire, *SPOT-25* stapesplasty outcome test 25

The following three predefined hypothesis were confirmed by our findings. First of all, the tinnitus, mental condition and social restrictions subscores correlated weakly with the postoperative air–bone gap, gain in air–bone gap and success defined as air–bone gap closure 10 dB or less. In addition, the correlation coefficient between the SPOT-25 total score and the postoperative GHSQ (r =  − 0.80) was at least 0.1 higher than between the SPOT-25 total score and the GBI (r =  − 0.28). The correlation coefficient between the SPOT-25 total score and the postoperative GHSQ (r =  − 0.80) was at least 0.2 higher than between the SPOT-25 total score and postoperative air conduction (r = 0.50), postoperative air–bone gap (r = 0.23), gain in air–bone gap (no correlation) and success defined as air–bone gap closure 10 dB or less (r =  − 0.23). Lastly, the mental condition and social restrictions subscores showed stronger correlations with the GHSQ, that is − 0.74 and − 0.71, respectively, than with the GBI, which had correlations of − 0.25 and − 0.24, respectively. Two predefined hypotheses were not confirmed by our findings. First, the correlation between the hearing function subscore and the GBI (r = -0.27) was not 0.1 higher than between the hearing function subscore and the GHSQ (r =  − 0.72). In addition, the correlation between the hearing function subscore and gain in air conduction (r =  − 0.36) was not 0.1 higher than the correlation between the hearing function subscore and postoperative air conduction (r = 0.55) nor 0.2 higher than the correlation between the hearing function subscore and the postoperative air–bone gap (r = 0.21) or success defined as air–bone gap closure to 10 dB or less (− 0.21). However, the correlation between the hearing function subscore and gain in air-conduction (r =  − 0.36) than with the air–bone gap (no correlation).

#### Measurement invariance

As hypothesized, the four-factor model fits best in our population of otosclerosis patients with a CFI of 0.833, a RMSEA of 0.087 and a SRMR of 0.091 as shown in Table 2. However, these results indicate a mediocre fit between the model and the data.

**Table 2:** Measurement invariance

Model	CFI	RMSEA (95% CI)	SRMR
4-factor model	0.833	0.087 (0.074–0.099)	0.091
3-factor model	0.637	0.127 (0.116–0.138)	0.109
2-factor model	0.624	0.129 (0.118–0.140)	0.106
1-factor model	0.574	0.137 (0.126–0.148)	0.105

*CFI* comparative fit index, *95% CI* 95% confidence interval, *RMSEA* root mean square error of approximation, *SRMR* standardized root mean square residual

#### Discriminative validity

The median translated SPOT-25 total score of the patient group was 46 (range: 14–80), while the healthy control group had a median score of 2 (range 0–14). The translated SPOT-25 total score and subscores of the patient group differed significantly from those of the healthy control group, indicating good divergent validity (*p*-value < 0.001).

#### Reliability

The translated SPOT-25 total score and subscores had Cronbach’s alphas of more than 0.70, which indicated good internal consistency.

A total of 100 patients completed the third measurement of the translated SPOT-25. The average time between the second and third measurement was 38 days. The intraclass correlation coefficients were more than 0.70 for the total score and the hearing function, mental condition and social restrictions subscores of the SPOT-25, which showed good test–retest reliability. Only the intraclass coefficient of the tinnitus subscore was 0.67, indicating lower test–retest reliability.

#### Responsiveness

The gain of the SPOT-25 scores correlated strongly with the gain of the GHSQ total score. Good or moderate correlations were found between the SPOT-25 subscores and the gain of the GHSQ total score. Regarding the audiometric results, only the total score and hearing function subscore of the SPOT-25 correlated moderately with gain of air conduction (Table 3). Three predefined hypotheses were confirmed by our findings. First, the correlation of change on the SPOT-25 with change on the GHSQ was at least 0.1 higher (r =  − 0.72) than the correlation of change on the translated SPOT-25 with gain in air conduction (r = 0.31) and at least 0.2 higher than the correlation of change on the translated SPOT-25 with the gain in air–bone gap (no correlation). In addition, the correlation of change in the hearing function domain with gain in air conduction (r = 0.39) was higher than the correlation of change on the translated SPOT-25 with the gain in air–bone gap (no correlation). Lastly, we found weak correlations between change in the tinnitus, mental condition and social restrictions subdomains and the gain in air–bone gap. One predefined hypothesis was not confirmed by our findings. The correlation of change on the translated SPOT-25 with gain in air conduction (r = 0.31) was not higher than the correlation of change on the translated SPOT-25 with the gain in air–bone gap (no correlation).

**Table 3:** Responsiveness

SPOT-25	Questionnaire	Audiometric results	
	Gain total score GHSQ	Gain AC	Gain ABG
Gain total score	** − 0.72**	**0.31**	0.14
Gain hearing function	** − 0.67**	**0.39**	**0.21**
Gain tinnitus	** − 0.47**	0.12	0.08
Gain mental health	** − 0.60**	0.16	** − **0.01
Gain social restrictions	** − 0.48**	**0.19**	0.05

Data is expressed as Pearson correlation coefficients. Correlation coefficients printed in bold were statistically significant with a p-value < 0.05

*ABG* air–bone gap, *AC* air conduction, *GHSQ* Glasgow Health Status Questionnaire, *SPOT-25* stapesplasty outcome test 25

## Discussion

### Summary of main results

In this study, the German version of the SPOT-25 was successfully forward–backward translated into the Dutch language without major discrepancies between translators. The translated SPOT-25 was easy to understand and easy to administer. We made sure the Dutch SPOT-25 was semantically and conceptually equivalent to the German version and it showed good validity, reliability and responsiveness.

### Translation and cultural adaptation

Translating a patient-reported outcome measure poses several problems, which can be contextual, translational and interpretational of nature. A contextual problem means that the translation of a question, which is based on country-specific conditions, leads to a question that is incomprehensible. An example of a translational problem is that the translation of a question leads to a longer and incomprehensible sentence. An interpretational problem means that a sentence can be translated in multiple ways, leading to a translated sentence that no longer has the same meaning as the original sentence [16]. These three problems were avoided by the 6-step process of translation and cross-cultural adaption used in this study. This process has been consolidated in literature and applied in several studies [17]. We chose an otorhinolaryngologist as forward translator 1 because an expert provides a more reliable translation from a measurement perspective. Forward translator 2 was a naive translator because he or she provides a translation that reflects the language of the Dutch population, as he or she is less influenced by the academic goal. Furthermore, the standardized translation and the involvement of patients in the process ensured the cultural adaptation of the translated SPOT-25 and that otosclerosis patients can understand the translated SPOT-25 in the intended way. The face-to-face evaluation was very useful to determine the correct use of the Dutch expressions and wording, as well as their complete understanding. For the pilot test, 15 patients with otosclerosis were included instead of 50 patients, because this was in accordance with the COSMIN checklist published at the time [13, 14, 17, 18].

### Validation

Hundred and thirty patients were included instead of 125 patients as described in the published protocol. The COVID-19 pandemic caused a loss to follow-up that was larger than expected. The included patients were unable to complete the postoperative measurements in the first months of the pandemic. In addition, we included three secondary care centers for patient inclusion, because less patients could undergo stapes surgeries in the tertiary care center due to the COVID-19 pandemic.

Test–retest reliability was assessed using the data from the two postoperative measurements. For reliability studies, a minimum of 50 patients is recommended. Even though a substantial amount of patient did not complete the third measurement, the remaining number was still sufficient. The first postoperative measurement (mentioned as the second measurement throughout this manuscript) was performed in clinic and the second postoperative measurement (mentioned as the third measurement throughout this manuscript) at home. We are aware that the different test setting is a potential confounder. However, having the second postoperative measurement take place at home was expected to limit loss to follow-up. The average time between the first postoperative and second postoperative measurement was 38 days, which was longer than expected. However, since stapes surgery yields stable audiometric results over the years, this extended time frame is unlikely to have affected the results, as hearing function would likely have remained stable [19].

Discriminative validity was assessed by using the data of the healthy control group. We wanted to make sure that the age of the included controls was approximately the same as that of the included otosclerosis patients. Based on three large cohort studies, otosclerosis patients are on average 39 to 47 years old when they undergo primary stapes surgery with an SD of 11–15 years [20–22]. Therefore, we chose to include controls in the age range 30–60 years only.

Using the confirmatory factor analysis, we verified that the four-factor model fits best to the data of otosclerosis patients. However, our results indicate a mediocre fit between the model and the data. Measurement invariance was not assessed in any of the published studies evaluating the validity of the SPOT-25 [7, 9–11].

### Tinnitus

The tinnitus subscore of the translated SPOT-25 cannot replace a tinnitus-specific questionnaire that measures the psychometric burden of tinnitus. The tinnitus subscore provides some preliminary data on the specific restrictions in daily life due to the impact of tinnitus. In a future study we could assess the correlation between the subscore tinnitus of the translated SPOT-25 and a tinnitus-specific questionnaire, such as the Tinnitus Handicap Inventory or Tinnitus Questionnaire [23, 24].

### Comparison with findings in the literature

Table 4 compares our results with the results of the original validation study of the SPOT-25 [7]. Our results were in line with the results of the original validation study. The Dutch SPOT-25 and German SPOT-25 both have a high internal consistency, good test–retest reliability and responsiveness. The postoperative tinnitus score and the postoperative audiometric results were not correlated in our study and in the original German validation study. These results are consistent with other studies analyzing the relation between tinnitus and postoperative audiometric results [25, 26]. Compared to the methods used for the translation, cultural adaptation and validation of the Danish, French, German and Greek SPOT-25, we employed a more comprehensive approach in line with the COSMIN guideline [7, 9–11, 13, 14]. We included more patients than the other studies, which evaluated between 35 and 56 participants. To assess construct validity and responsiveness, participants also completed additional quality of life questionnaires, i.e. the GBI and GHSQ. We predefined hypotheses about the correlations between the scores of these questionnaires and published them in the study protocol. In addition, we are the first to assess the measurement invariance of the SPOT-25.

**Table 4:** SPOT-25 scores of the current study and the original validation study

Score SPOT-25	Current study (n = 115)			Original validation study (n = 52)		
	Preoperative (SD)	Postoperative (SD)	Gain* (SD)	Preoperative (SD)	Postoperative (SD)	Gain* (SD)
Total score	48 (15)	27 (19)	**21 (19)**	50 (17)	26 (19)	24 (21)
Hearing function	59 (14)	32 (23)	**27 (24)**	60 (20)	30 (24)	30 (24)
Tinnitus	40 (30)	29 (25)	**12 (27)**	42 (33)	32 (28)	11 (25)
Mental condition	41 (18)	23 (20)	**18 (20)**	44 (18)	21 (19)	23 (22)
Social restrictions	37 (22)	19 (19)	**17 (21)**	43 (21)	21 (18)	20 (25)

*n* number, *SD* standard deviation, *SPOT-25* stapesplasty outcome test 25

*Tested using the paired students t-test. Gains printed in bold were statistically significant with a p-value < 0.001

Our research showed a good correlation between subscore hearing function and the postoperative air conduction. In line with the literature, the total score and subscores of the translated SPOT-25 did not correlate well with surgical success (ABG closure ≤ 10 dB) [4]. Consequently, the postoperative air–bone gap is an inadequate tool to reflect psychosocial aspects of disease-specific quality of life. In our opinion, the SPOT-25 could be implemented as an additional outcome measure to improve otosclerosis research and clinical practice. For example, the implementation of the translated SPOT-25 enables us to consider the health-related quality of life in otosclerosis patients after primary stapes surgery. This tool helps us consider the patient’s emotional well-being and improves how ENT surgeons communicate the expected outcomes of stapes surgery to both patients and their families.

## Conclusion

In this study we translated and culturally adapted the SPOT-25 into Dutch and validated the translated questionnaire in a Dutch otosclerosis patient group. The Dutch SPOT-25 showed good validity, reliability and responsiveness and can be implemented as an additional outcome measure to improve the otosclerosis research and clinical practice. The Dutch SPOT-25 enables the otorhinolaryngologist to take health-related quality of life into account when deciding on what surgical technique to use during primary stapes surgery and provides an additional outcome measure after stapes surgery.

## Supplementary Information

Below is the link to the electronic supplementary material.

Supplementary file1 (DOCX 22 kb)

## Data Availability

The questionnaire is open access. The data is available upon request.
