# Supplementary material for: Translation, cultural adaptation and validation of the Stapesplasty Outcome Test 25 (SPOT-25) for measurement of disease-specific health-related quality of life in Dutch otosclerosis patients: a prospective study
Source: Eur Arch Otorhinolaryngol. 2025 May 8;282(9):4477–86. doi: 10.1007/s00405-025-09353-5 (PMC12423205; doi:10.1007/s00405-025-09353-5)
Supplement: Supplementary file 1 — Supplementary file1 (DOCX 22 kb) [file 405_2025_9353_MOESM1_ESM.docx]

| Item 4 |  |
| --- | --- |
| German | Ich habe Schwierigkeiten, jemanden aus größerer Entfernung zu verstehen. |
| Forward translation 1 | Ik heb moeilijkheden om iemand, die op grotere afstand staat, te verstaan. |
| Forward translation 2 | Ik vind het moeilijk om iemand op grotere afstand te verstaan. |
| Synthesis – Dutch version SPOT-25 (1) | Ik vind het moeilijk om iemand, die op grotere afstand staat, te verstaan. |
| Backward translation 1 | Ich finde es schwierig jemanden, der weit entfernt steht, zu verstehen |
| Backward translation 2 | Es fällt mir schwer Menschen zu verstehen, die weit weg stehen |
| Expert Committee Review – Prefinal Dutch version SPOT-25 (3) | Ik vind het moeilijk om iemand, die op grotere afstand staat, te verstaan. |

**Appendix 1**

Example of the translation process of one item (question 4) from the Stapesplasty Outcome Test 25

**Appendix 2**

Stapesplasty Outcome Test 25 comparisons

| Original German version | Translated Dutch version |
| --- | --- |
| 1. Hörverlust | 1. Gehoorverlies |
| 2. Sprache klingt gedämpft und undeutlich | 2. Spraak klinkt gedempt en onduidelijk |
| 3. Ich habe Schwierigkeiten beim Erkennen der Richting, aus der das Geräusch kommt | 3. Ik vind het moeilijk om te herkennen uit welke richting een geluid komt |
| 4. Ich habe Schwierigkeiten, jemanden aus größerer Entfernung zu verstehen | 4. Ik vind het moeilijk om iemand, die op grotere afstand staat, te verstaan |
| 5. Ich überhöre das Telefon oder den Wecker | 5. Ik hoor de telefoon of wekker niet afgaan |
| 6. Ich habe Schwierigkeiten, etwas in lauter Umgebung zu verstehen | 6. Ik vind het moeilijk om iets te verstaan in een rumoerige omgeving |
| 7. Ich habe Schwierigkeiten beim Verstehen, wenn mehrere Leute gleichzeitig sprechen | 7. Ik vind het moeilijk om iemand te verstaan wanneer meerdere mensen tegelijkertijd praten |
| 8. Ich habe Schwierigkeiten mit dem Verstehen beim Telefonieren | 8. Ik vind het moeilijk om iemand aan de telefoon te verstaan |
| 9. Ich habe Schwierigkeiten beim Musikhören, Fernsehen | 9. Ik heb moeite met het beluisteren van muziek en televisie |
| 10. Ich habe Schwierigkeiten bei der Kommunikation mit Menschen, die mein Hörproblem nicht kennen | 10. Ik vind het moeilijk om te communiceren met mensen die niet op de hoogte zijn van mijn hoorprobleem |
| 11. Ohrgeräusche (Pfeifen, Rauschen) | 11. Oorsuizen (fluittoon, ruis) |
| 12. Durch meine Ohrgeräusche habe ich Schwierigkeiten, andere zu verstehen | 12. Door mijn oorsuizen heb ik moeite om anderen te verstaan |
| 13. Meine Ohrgeräusche beeinträchtigen mich in alltäglichen Situationen mit vorhandenen Nebengeräuschen | 13. Mijn oorsuizen beperkt mij in dagelijkse situaties waarbij ook omgevingsgeluiden voorkomen |
| 14. Meine Ohrprobleme machen mich antriebslos/mindern meine Motivation | 14. Door mijn gehoorprobleem ben ik minder gedreven; ben ik minder gemotiveerd |
| 15. Wegen des Hörverlusts habe ich Angst, andere falsch zu verstehen | 15. Door mijn gehoorverlies ben ik bang dat ik anderen verkeerd versta |
| 16. Der Hörverlust führt zu Situationen, die mir peinlicht sind | 16. Het gehoorverlies brengt mij in situaties die voor mij pijnlijk zijn |
| 17. Ich sorge mich, dass meine Ohrpobleme in Zukunft zunehmen | 17. Ik ben bezorgd dat mijn gehoorprobleem in de toekomst toeneemt |
| 18. Das Tragen von Hörgeräten führt dazu, dass mein Hörproblem für andere sichtbar wird | 18. Het dragen van een gehoorapparaat leidt ertoe dat anderen kunnen zien dat ik een gehoorprobleem heb |
| 19. Angst vor einer Operation oder Nachoperation | 19. Ik ben bang voor een operatie of heroperatie |
| 20. Aufgrund des Hörproblems bin ich zurückhaltend bei der Knüpfung neuer Bekanntschaften/Freundschaften | 20. Door mijn gehoorprobleem ben ik terughoudend in het aangaan van nieuwe vriendschappen of relaties |
| 21. Aufgrund der Ohrprobleme habe ich Probleme im Beruf bzw. Bei Tätigkeiten in der Öffentlichkeit (Erledigungen bei Behörden, Einkaufen etc.) | 21. Door mijn gehoorprobleem heb ik problemen met het functioneren op het werk en/of dagelijkse beslommeringen (contact met overheid, boodschappen doen, e.d.) |
| 22. Aufgrund des schlecgten Hörens fühle ich mich sehr angestrengt | 22. Door mijn slechte gehoor voel ik me zeer vermoeid |
| 23. Einschränkung der beruflichen Leistungsfähigkeit | 23. Beperking in mijn beroepsuitoefening |
| 24. Das Tragen eines Höregeräts würde mich beeinträchtigen (beruflich, privat, sportlich) | 24. Het dragen van een gehoorapparaat brengt voor mij beperkingen met zich mee (berope, privé, sport) |
| 25. Gesamteinschätzung der Beeinträchtigung der Lebensqualität durch die Ohrerkrankung | 25. Algemene oordeel over de invloed van het gehoorverlies op de kwaliteit van leven |
